# Sixty-day oral antioxidant supplementation enhances sperm morphology and seminal plasma antioxidant capacity in stallions

**DOI:** 10.1093/jas/skag265

**Published:** 2026-08-25

**Authors:** Chiara Del Prete, Consiglia Longobardi, Valentina Longobardi, Federica Piscopo, Roberto Ciarcia, Natascia Cocchia, Maria P Pasolini, Monica I Cutrignelli, Alessandro Vastolo

**Affiliations:** Department of Human Science, Link Campus University, Rome 00165, Italy; Department of Veterinary Medicine and Animal Production, University of Naples Federico II, Naples 80137, Italy; Department of Experimental Medicine, University of Rome—Tor Vergata, Rome 00133, Italy; Department of Veterinary Medicine and Animal Production, University of Naples Federico II, Naples 80137, Italy; Department of Veterinary Medicine and Animal Production, University of Naples Federico II, Naples 80137, Italy; Department of Veterinary Medicine and Animal Production, University of Naples Federico II, Naples 80137, Italy; Department of Veterinary Medicine and Animal Production, University of Naples Federico II, Naples 80137, Italy; Department of Veterinary Medicine and Animal Production, University of Naples Federico II, Naples 80137, Italy; Department of Veterinary Medicine and Animal Production, University of Naples Federico II, Naples 80137, Italy

**Keywords:** dietary supplementation, oxidative stress, equine, spermatozoa, semen quality

## Abstract

While dietary supplementation with antioxidants has been identified as an effective strategy for mitigating oxidative stress (OS) damage in stallion sperm, specific protocols and the duration of its efficacy remain poorly defined. This study aimed to evaluate the effects of a 60-d dietary supplementation with a commercial antioxidant combination on semen quality and OS biomarkers in stallions. Stallions were divided into two groups: the control group (*n *= 5; CTRL) received 30 g/500 kg of placebo while the treated group (*n *= 5; OXY) received 30 g/500 kg of Oxyliver daily for 60 d. Semen was evaluated for volume, concentration, viability, morphology, membrane integrity, motility, and kinetic parameters every 15 d for the 60-d supplementation period and for an additional 60 d after treatment cessation (D0, D15, D30, D45, D60, DP15, DP30, DP45, DP60). Total antioxidant capacity (TAC), ferric reducing/antioxidant power (FRAP), and malondialdehyde (MDA) were measured at 30-d intervals. The change in sperm parameters over time differed significantly between groups, specifically for sperm membrane integrity (*P* = 0.004), total motility (*P* = 0.003), morphological abnormalities (*P* = 0.028), and OS parameters (TAC *P* = 0.031, FRAP *P* = 0.044, MDA *P* = 0.048). Compared to CTRL, the OXY group showed greater sperm membrane integrity (*P* = 0.004) and total motility (*P* = 0.039) at D60. Conversely, total morphological abnormalities were lower in OXY at D60 (*P* = 0.003), DP15 (*P* = 0.009), and DP30 (*P* = 0.018), mainly due to a reduction in tail defects at the same time points (*P* = 0.008, *P* = 0.014, *P* = 0.020, respectively). Moreover, TAC and FRAP were greater in the OXY group at D30 (*P* = 0.004 and *P* = 0.012, respectively) and D60 (*P* = 0.001 and *P* = 0.009, respectively), while MDA concentrations were lower in the OXY group at D60 (*P* < 0.001), DP30 (*P* = 0.033) and DP60 (*P* = 0.003). In conclusion, despite the small sample size, this longitudinal study provides preliminary evidence that a 60-d supplementation with this combined formula enhances seminal plasma antioxidant capacity during supplementation and reduces lipid peroxidation even after the end of supplementation. It also induces a transient improvement in sperm membrane functionality and total motility at specific time points, whereas sperm morphology showed a more robust improvement beyond the supplementation period.

## Teaser

A 60-d antioxidant supplementation improves sperm quality and enhances seminal antioxidant capacity in stallions, with beneficial effects persisting beyond treatment. These findings support a practical strategy to mitigate oxidative stress and optimize sperm parameters in breeding stallions.

## Introduction

Oxidative stress (OS) has been considered a major contributory factor to compromised male fertility (Pintus and Ros-Santaella 2021). OS occurs when the generation of reactive oxygen species (ROS) exceeds the capacity of endogenous antioxidant systems to neutralize them (Kaltsas 2023). High levels of ROS are commonly produced in the male reproductive system, as reproduction is an energetically demanding process that increases metabolic rate (Blount et al. 2016). This condition is particularly relevant in stallions, where OS has been identified as a key factor influencing semen quality and fertility outcomes, especially under intensive breeding management (Ullah et al. 2025). Moreover, in this context, the testes appear to be more vulnerable to oxidative damage than other tissues, and spermatozoa are particularly vulnerable to oxidative damage due to their inherently limited antioxidant defenses and the high concentration of polyunsaturated fatty acids (PUFA) in their plasma membrane (García et al. 2011; Simmons et al. 2018). In stallions, this intrinsic susceptibility is further exacerbated by routine semen-handling procedures, such as dilution, cooling, and transport, which can increase ROS production and shorten sperm lifespan (de Oliveira et al. 2017). Despite this susceptibility, certain concentrations of ROS are essential for specific physiological processes critical to fertilization, including sperm capacitation, acrosome reaction, and binding to the zona pellucida (Baumber et al. 2003). Excessive or dysregulated ROS compromise all spermatic compartments and functions, notably impairing sperm quality parameters with consequential effects on embryonic development (Peña et al. 2019).

Antioxidant dietary supplementation is widely regarded as an effective strategy in both subfertile and healthy males to counteract OS in the reproductive tract and enhance the antioxidant profile in seminal plasma (Ribas-Maynou and Yeste 2020; Pintus and Ros-Santaella 2021; Torres-Arce et al. 2021; Dimitriadis et al. 2023; Ullah et al. 2025). In stallions, although evidence is more limited, similar beneficial effects have been reported (Contri et al. 2011; Del Prete et al. 2018). This beneficial effect is mediated by both enzymatic antioxidants, such as superoxide dismutase (SOD), and non-enzymatic antioxidants, including vitamins C and E, which act synergistically to maintain redox balance and protect spermatozoa from oxidative damage (Bouhadana et al. 2025). Indeed, this strategy is effective in improving exogenous antioxidant defenses and preventing OS-associated sperm damage (Contri et al. 2011; Del Prete et al. 2018). Greater-quality semen may better preserve its functional properties during cooling or freezing, thereby improving fertility rates in artificial insemination programs (Heckenbichler et al. 2011).

In literature, only a few studies have investigated the use of botanicals with antioxidant properties or enzymatic and non-enzymatic antioxidants, alone or in combination with compounds such as selenium and PUFAs in stallions (Deichsel et al. 2008; Contri et al. 2011; Freitas et al. 2016; Del Prete et al. 2018; Bazzano et al. 2021; Puerta et al. 2024). These studies consistently demonstrated improvements in sperm motility, membrane and acrosome integrity, and the antioxidant capacity of seminal plasma after 6–12 wk of supplementation, particularly when using a combination of antioxidants (Deichsel et al. 2008; Freitas et al. 2016; Bazzano et al. 2021; Puerta et al. 2024). However, long-term effects after the end of supplementation remain largely unexplored, leaving a gap in understanding the duration and persistence of antioxidant benefits in stallions.

The optimal antioxidant supplementation in stallions has yet to be identified. Using an authorized, horse-specific commercial product containing a combination of antioxidants offers a reliable, standardized approach for breeding stallions, backed by manufacturer-documented safety and prior findings (Del Prete et al. 2024, 2026). Additionally, such a multi-ingredient formula is hypothesized to leverage the potential synergistic action of multiple antioxidants to provide broad-spectrum protection against OS (Liu 2003; Fragopoulou et al. 2018). The commercial product used in this study is Oxyliver, a complementary antioxidant feed designed to support proper metabolic function and promote liver health. It contains silybin, which acts as an antioxidant and can stimulate the liver’s endogenous antioxidant production, including glutathione and SOD (Surai 2015; Cai et al. 2024). It also includes melon extract, a natural source of SOD, as well as curcumin, vitamins C and E.

While previous studies in stallions have documented the beneficial effects of dietary antioxidants on semen parameters during the supplementation period, including improvements in sperm motility, membrane and acrosome integrity, and seminal plasma antioxidant capacity (Deichsel et al. 2008; Contri et al. 2011; Freitas et al. 2016; Del Prete et al. 2018; Bazzano et al. 2021), the durability of these effects after cessation of supplementation remains largely unexplored. Therefore, the present study aimed to address this knowledge gap by evaluating semen quality and OS biomarkers not only during the supplementation period but also after treatment withdrawal. The effects of 60-d dietary supplementation covering the entire spermatogenesis cycle in stallions were evaluated using a commercial antioxidant combination on semen quality and OS biomarkers. It is hypothesized that antioxidant supplementation could induce a sustained carry-over effect beyond the supplementation period, thereby maintaining the antioxidant status of seminal plasma and protecting spermatozoa from oxidative damage with potential benefits for sperm viability, morphology, and motility.

## Materials and methods

The Ethical Animal Care and Use Committee of the Federico II University of Naples approved experimental design and animal treatments (PG/2025/0015958, 06/02/2025).

### Experimental design

Ten stallions of different breeds (Salernitano, Italian heavy draft horse, and Haflinger) aged between 5 and 18 yr (median age 14 yr) were involved in this study. Stallions were housed in individual box stalls at the breeding station of the Regional Horse Breeding Center of Santa Maria Capua Vetere (Caserta, Italy) under natural photoperiod and ambient temperature conditions, maintaining uniform housing and environmental conditions throughout the study period.

Stallions were divided into a control group (CTRL; *n *= 5) and a treated group (OXY; *n *= 5). To ensure a breed balance, the eight Salernitano stallions were randomly allocated (four to each group), while the remaining two stallions (one Haflinger and one Italian Heavy Draft) were assigned one to the CTRL group and one to the OXY group, respectively. Following this allocation, we verified that the two groups were homogenous, with no significant differences regarding age (median age: 11.8 yr in the CTRL group and 13.2 yr in the OXY group) and body weight (514 [455–600] kg median [range] weight kg in CTRL vs. 549 [490–594] kg OXY group). Each stallion in the OXY group received a 30 g/500 kg measuring cup of Oxyliver (Candioli Pharma, Turin, Italy) per day (d), while stallions in the CTRL group received a placebo in the same amount and manner. The supplementation was administered daily for 60 d (from February to April), during which semen samples were collected every 15 d; sampling continued at the same intervals for an additional 60 d after the end of supplementation (from April to June). Regarding semen collection management, all stallions followed the same routine schedule during the study period: semen was collected from each stallion 1 wk before the onset of the study and then once a week throughout the study, either for the research analyses or for breeding purposes. For each time point, one ejaculate was collected and analyzed per stallion.

At each evaluation time point (D0; D15; D30; D45; D60; DP15; DP30; DP45; DP60), semen samples were assessed immediately after collection for volume, concentration, viability, morphology, membrane integrity, motility, and kinetic parameters. Additionally, OS status in seminal plasma was characterized by assessing antioxidant capacity using the total antioxidant capacity (TAC), and the ferric reducing/antioxidant power (FRAP) assay, together with malondialdehyde (MDA) concentration as an indicator of lipid peroxidation. These parameters were evaluated every 30 d throughout the study period to investigate the effects of the supplement on seminal plasma redox balance.

### Diet and antioxidant supplementation

Stallions were maintained under the same feeding and management conditions throughout the study period. Water and mixed hay were offered ad libitum. Mixed hay consisted of approximately 80–90% grass hay (ryegrass and fescue) and 10–20% alfalfa hay. In addition, stallions received a commercial pelleted concentrate twice daily, based on body weight and maintenance requirements. Daily concentrate average intake was 2.8 ± 0.56 kg/horse/d. No dietary changes were introduced during the study period, and no additional vitamin, antioxidant, or nutraceutical supplements were provided. The commercial concentrate consisted of durum wheat middling, flaked corn, oats, barley flour, flaked barley, soybean meal, calcium carbonate, cane molasses, flaked faba beans, carob seeds, soybean oil and fats, and sodium chloride. The concentrate also provided standard vitamin and trace mineral supplementation according to commercial feeding practices for breeding stallions, including vitamin E, selenium, zinc, copper, and manganese. The proximate chemical composition (% Dry Matter) of the mixed hay and concentrate is reported in Table 1.

**Table 1:** Proximate chemical composition of mixed hay (MH) and commercial concentrate (CC; % dry matter basis).

Item	DM, %	CP, %	NDF, %	ADF, %	ADL, %	EE, %	Ash, %	Starch, %
**MH**	91.5	6.73	57.8	39.2	12.2	1.34	9.20	18.4
**CC**	87.0	13.5	21.6	10.6	1.5	3.98	5.98	42.2

DM, dry matter; CP, crude protein; NDF, neutral detergent fiber; ADF, acid detergent fiber; ADL, acid detergent lignin; EE, ether extract.

The antioxidant supplementation used in this study was Oxyliver (Candioli Pharma, Turin, Italy). Stallions in the OXY group received Oxyliver once daily at a dose of 30 g/500 kg BW mixed with the morning concentrate ration, while stallions in the CTRL group received the placebo in the same amount and manner. Supplement and placebo intake were visually monitored daily by farm personnel, and no refusals were observed during the study period. The placebo consisted of 100% maltodextrin and was matched to Oxyliver for color, odor, consistency, appearance, and feeding method to ensure comparable voluntary intake and handling conditions between groups. All personnel involved in the study (farm staff, semen collectors, semen evaluators) were completely blinded to the treatment group allocation throughout the study.

Oxyliver is a veterinary liver-support supplement formulated for horses, composed of maltodextrin, fructooligosaccharides, phosphatidylcholine, soya protein concentrate, products and by-products of fruit processing (melon extract; Melofeed® Lallemand Animal Nutrition: natural SOD source), vitamin C, vitamin E, Choline chloride, Citrus sinensis L., Curcuma longa L., Silybum marianum L. glycine, methionine, colloidal silica, lecithin, and microcrystalline cellulose. The micronutrient composition of CTRL and OXY based on the feeding rate of 30 g/500 kg BW/d is reported in Table 2.

**Table 2:** Micronutrients intake.

Additives	CTRL, mg/kg d	OXY, mg/kg d
**Vitamin A**	12.99	17.632
**Vitamin D3**	0.11	0.152
**Biotin**	1.40	1.9
**Choline chloride**	280.00	380
**Folic Acid**	5.60	7.6
**Niacinamide**	70.00	95
**Calcium D-pantothenate**	22.40	30.4
**Vitamin B1 (thiamine)**	25.20	34.2
**Vitamin B12**	0.084	0.114
**Vitamin B2 (riboflavin)**	19.60	26.6
**Vitamin B6 (pyridoxine)**	16.80	22.8
**Vitamin E**	280	380
**Vitamin K3 (menadione)**	28.00	38
**Copper sulfate**	42.00	57
**Iodine**	2.52	3.42
**Iron**	294	399
**Manganese**	252	342
**Selenium**	1.12	1.52
**Zinc**	157	213

### Semen collection and evaluation

A mare exhibiting behavioral signs of estrus was used as a teaser to stimulate the stallions during semen collection. Semen was collected using a pre-warmed, lubricated Missouri-type artificial vagina and immediately filtered through a semen filter pouch (Minitube, Tiefenbach, Germany). All materials used for semen collection were maintained in a thermostatic chamber at 37 °C until assembly to minimize thermal shock to the spermatozoa. Immediately after semen collection, the volume of raw semen was measured using 50 mL graduated tubes (Corning, New York, USA), and sperm concentration was determined with a Sperm-Cue photometer (Minitube, Tiefenbach, Germany). At the same time, a rapid subjective assessment of mass sperm motility was performed. Aliquots of the fresh, undiluted semen were then collected for the assessment of morphology, viability, and membrane integrity. A portion of the raw semen (10 mL) was centrifuged at 1,500 × g for 10 min to separate seminal plasma from spermatozoa, and the plasma was stored at −80 °C until antioxidant evaluation.

Semen samples were then diluted with a commercial extender (INRA96, IMV Technologies, L’Aigle, France) to achieve a final concentration of 50 × 10⁶ spermatozoa/mL and transported at refrigeration temperature (5 °C) to the laboratory for the assessment of motility and kinetic parameters using the Sperm Class Analyzer system (SCA, Microptic, Barcelona, Spain) within 1 h from collection. To minimize bias, all semen parameter evaluations were performed under blinded conditions with respect to treatment and time point.

### Evaluation of sperm viability and morphology

Sperm viability and morphology were assessed using eosin staining (Europath, Avellino, Italy), which allows simultaneous evaluation of membrane integrity and morphological defects (Murcia-Robayo et al. 2018). Live spermatozoa remained unstained due to intact plasma membranes, whereas dead spermatozoa appeared pink as eosin penetrated compromised membranes. Morphological abnormalities recorded included bent or coiled tails, midpiece defects, cytoplasmic droplets, and total abnormality percentage. For staining, 5 μL of semen was mixed with 10 μL of eosin, smeared on a slide, and air-dried. A total of 200 spermatozoa per sample were examined under a light microscope (Eclipse E200, Nikon, Tokyo, Japan) at 400× magnification by a single trained operator, and percentages were calculated.

### Sperm membrane functionality

Sperm membrane functionality was assessed by the hypoosmotic swelling (HOS) test, as previously described (Domínguez-Rebolledo et al. 2010). One hundred μL of semen were mixed with 1 mL of a hypo-osmotic solution (0.73 g sodium citrate and 1.35 g fructose in 100 mL of distilled water, 150 mOsm) and incubated at 37 °C for 30 min. A drop of diluted semen was placed on a clean slide and covered with a cover slip. A total of 200 spermatozoa were counted in different fields (magnification: ×100) under a phase contrast microscope (E200 Nikon, Tokyo, Japan), and the percentage of spermatozoa positive to the HOS test (HOS+, having coiled tails), ie those with intact membranes, was determined. Spermatozoa exhibiting tail and neck morphological defects were excluded from the HOS + fraction to calculate the percentage of functionally intact sperm.

### Sperm motility and kinetic parameters

Sperm motility was assessed by a SCA (Microptic SL, Veterinary Edition, Barcelona, Spain) installed on a camera-equipped light microscope system (Eclipse E200, Nikon, Tokyo, Japan) fitted with a warmer stage. The following parameters were considered: total motility (%), progressive motility (%), the percentage of sperm subpopulations (rapid, medium, slow, and immotile spermatozoa), average path velocity (VAP; μm/s), straight-line velocity (VSL; μm/s), curvilinear velocity (VCL; μm/s), straightness (STR; %), linearity (LIN; %), amplitude of lateral head displacement (ALH, beats/s), beat cross-frequency (BCF, beats/s), and hyperactive (%). SCA system settings for stallion semen classified as spermatozoa, all the particles sized between 5 and 80 μm^2^, and as progressively motile spermatozoa with 75% STR. Spermatozoa were classified based on VCL into rapid (>100 μm/s), medium (65–100 μm/s), slow (10–65 μm/s), and static (<10 μm/s). Sixty frames per second with a minimum contrast of 35 were acquired. For the assessment, an aliquot of semen was incubated at 37 °C for 10 min, after which 3 μL of semen were loaded onto a pre-warmed glass microscope Leja slide (Leja, 4-chamber counting slides; 20 μm nominal chamber depth, Leja Products BV, Nieuw-Vennep, The Netherlands). At least 500 sperm cells in five randomly selected fields were evaluated.

### Evaluation of oxidative status

Seminal plasma aliquots intended for oxidative-status analyses were immediately stored at −80 °C after collection and thawed only once, immediately before analysis. Samples collected from the same stallion at the different experimental time points were analyzed in duplicate within the same analytical run.

### TAC assay

TAC was evaluated by a commercial TAC assay kit (ab65329 Abcam, Cambridge, UK), following the manufacturer’s instructions. In particular, 100 µL of seminal plasma (diluted 1:500) were added to a 96-well plate, followed by the addition of 100 µL of Cu^2+^ working solution. The plate was then incubated at room temperature for about 30 min in the dark on an orbital shaker, and the absorbance was immediately read at 570 nm using a spectrophotometer (Thermo Fisher Scientific, Rodano, Italy). TAC values in the samples were calculated based on a Trolox standard curve. The mean intra-assay coefficient of variation (CV) was 2.47%.

### FRAP assay

FRAP assay on animals’ seminal plasma was performed using a commercial FRAP assay kit (MAK509; Sigma-Aldrich, Milano, Italy), following the manufacturer’s instructions. Briefly, 50 µL of seminal plasma (diluted 1:10) were transferred into a flat-bottom 96-well plate, and 200 µL of working reagent containing Fe3+ were added to each well. After 40 min of incubation at room temperature, absorbance was measured at 610 nm using a spectrophotometer (Thermo Fisher Scientific, Rodano, Italy). The samples’ ferric reduction potential was quantified according to a standard curve prepared with Fe^2+^ solution (1.8 mM). The mean intra-assay CV was 3.37%.

### Evaluation of lipid peroxidation

Lipid peroxidation was measured according to the concentration of MDA, following the method described by Ohkawa et al. (1979) and applied to stallion seminal plasma by Wolkmer et al. (2019). Seminal plasma samples were first diluted with distilled water. Subsequently, 100 µL of 20% acetic acid, 100 µL of 0.8% thiobarbituric acid (TBA, Sigma-Aldrich, Milano, Italy), and 40 µL of 8.1% sodium dodecyl sulfate (Bio-Rad Hercules, California, USA) were added. Samples were incubated at 100 °C for 2 h, and aliquots, after centrifugation, were transferred to a 96-well microplate reader and read at 532 nm using a spectrophotometer (Thermo Fisher Scientific, Rodano, Italy). A calibration curve was prepared using a standard solution of MDA.

### Statistical analysis

Statistical analysis was performed using SPSS software (version 30.0; SPSS Inc., Chicago, IL. The stallion was considered the experimental unit. Normality was assessed using the Shapiro–Wilk test and visual inspection of normal Q–Q plots of the residuals, while homoscedasticity was verified through the evaluation of residual-vs.-predicted value plots. To satisfy these assumptions, percentage-based variables (eg, sperm motility and morphological abnormalities) were subjected to an arcsine square root transformation, while absolute value parameters (volume, concentration, sperm kinetic parameters, OS biomarkers) were log-transformed (log_10_) before analysis. Data were analyzed using a Linear Mixed Model for repeated measures. In the model, treatment group, time, and their interaction (Group × Time) were defined as fixed effects and the individual stallion as a random effect. An autoregressive (AR1) covariance structure was used to model the repeated measurements over time. When a significant overall interaction (Group × Time) was found, subsequent post-hoc pairwise comparisons between groups within specific time points were performed, applying Bonferroni correction for multiple testing. For all statistically significant post-hoc pairwise differences, the estimated mean differences ± standard error (SE) were reported. All statistical models were run on transformed datasets, whereas non-transformed mean values ± SE are reported in the text, tables, and figures for biological clarity. Statistical significance was set at *P* ≤ 0.05, with a statistical trend declared for biologically relevant differences at *P *< 0.10).

## Results

In the present study, no concentrate or supplement refusals were observed during diet supplementation in both groups. All stallions completed the study, and no complications or side effects related to the diet supplementation were observed. Moreover, no ejaculates were excluded from the study, and all stallions successfully contributed samples at all designated time points.

### Semen quality parameters

No significant effects of treatment group, time, or Group × Time interaction were observed for semen volume or sperm concentration (*P* > 0.05). Mean values calculated across all evaluation time points were comparable between CTRL and OXY groups (61.9 ± 4.0 vs. 69.2 ± 8.4 mL for the CTRL and OXY groups, respectively), and sperm concentration (224.0 ± 8.7 × 10⁶ spermatozoa/mL vs. 209.3 ± 14.0 × 10⁶ spermatozoa/mL for the CTRL and OXY groups, respectively; Table S1).

The results on sperm viability and sperm membrane functionality are reported in Table 3. No main effect of Sperm viability remained high throughout the experimental period, showing no significant treatment effect at any of the time points.

**Table 3:** Sperm viability and sperm membrane functionality (HOS+) at different time points between control (CTRL) and treated (OXY) groups in stallions.^1^

	Viability, %			HOS+, %	
	CTRL	OXY		CTRL	OXY
D0	92.2 ± 3.7	94.2 ± 4.1		59.8 ± 5.3	61.7 ± 5.3
D15	90.0 ± 5.3	83.5 ± 6.0		55.8 ± 5.3	54.5 ± 5.5
D30	87.2 ± 2.7	92.8 ± 3.0		59.7 ± 5.3	70.7 ± 5.3
D45	92.4 ± 3.2	91.2 ± 3.6		68.1 ± 5.3	71.8 ± 5.3
D60	91.2 ± 2.0	97.2 ± 2.3		60.2 ± 5.3^a^	82.6 ± 5.3^b^
DP15	95.0 ± 2.9	93.0 ± 3.2		67.2 ± 5.3	77.4 ± 5.3
DP30	95.6 ± 3.0	93.5 ± 3.3		69.6 ± 5.3	76.8 ± 5.3
DP45	93.2 ± 2.0	90.8 ± 2.5		68.4 ± 5.3	71.0 ± 5.3
DP60	94.8 ± 1.5	98.8 ± 1.7		70.4 ± 5.3	78.2 ± 5.3

1 Data are expressed as mean ± SEM.

a,b Indicates significant differences between groups at a specific time point; *P *< 0.05.

Sperm membrane functionality was also generally high; however, a highly significant group × time interaction was confirmed (*P* = 0.004), demonstrating that the chronological pattern of membrane integrity differed significantly between the groups (CTRL vs. OXY) over the storage period, in particular sperm membrane integrity was significantly greater in the OXY group compared to the CTRL group on D60 (non-transformed mean differences 22.4 ± 7.55%; *P* = 0.004). No statistically significant differences between the groups were observed at any other time point during the study.

**Figure 1:**
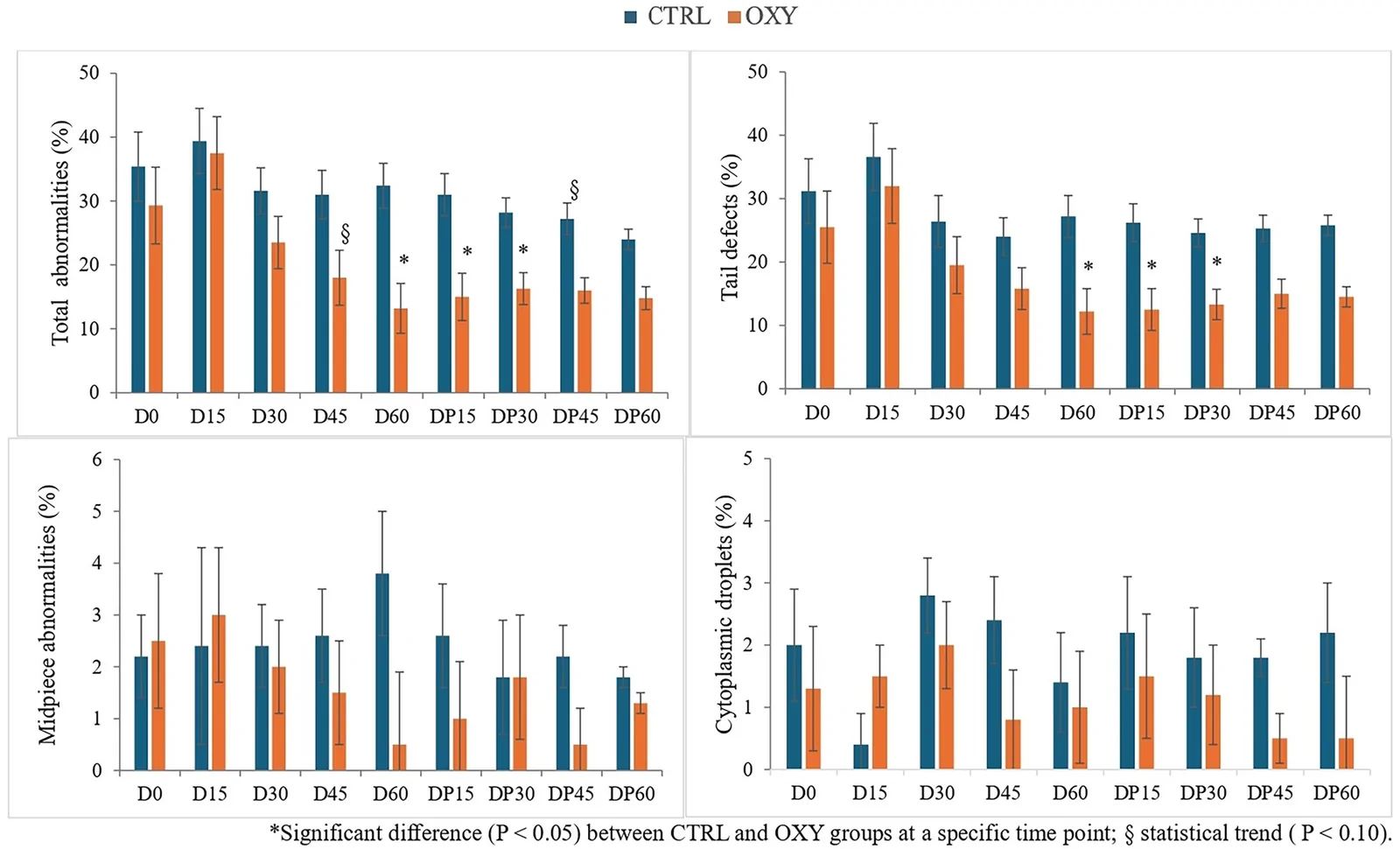
Percentage of total sperm morphological abnormalities, tail defects, midpiece abnormalities, and cytoplasmic droplets in the control (CTRL) and treated (OXY) groups at different evaluation times in stallions. Data are expressed as mean ± SEM.

The results of sperm morphological abnormalities are reported in Figure 1 and in Table S2. Sperm abnormalities were significantly affected by group × time interaction (*P* = 0.018), confirming that the treatment distinctly modulated the progression of morphological defects over time. A strong protective effect of the treatment has been demonstrated by a significantly lower percentage of total defects in the OXY group at D60 (non-transformed mean difference: 17.60 ± 5.35%; *P* = 0.003), DP15 (non-transformed mean difference: 14.80 ± 5.35%; *P* = 0.009), and DP30 (non-transformed mean difference: 12.80 ± 5.35%; *P* = 0.018). Moreover, a trend toward a lower percentage of defects in the OXY group was also noted at D45 (*P* = 0.052) and DP60 (*P* = 0.053).

In particular, the percentage of sperm displaying tail defects was significantly lower in the OXY group compared to the CTRL group on D60 (non-transformed mean difference: 13.8 ± 5.03%; *P* = 0.008), DP15 (mean difference: 12.4 ± 5.03%; *P* = 0.014), and DP30 (mean difference: 11.60 ± 5.03%; *P* = 0.020). Conversely, no significant differences between groups were observed for midpiece and cytoplasmic droplet abnormalities.

### Sperm motility and kinetic parameters

**Figure 2:**
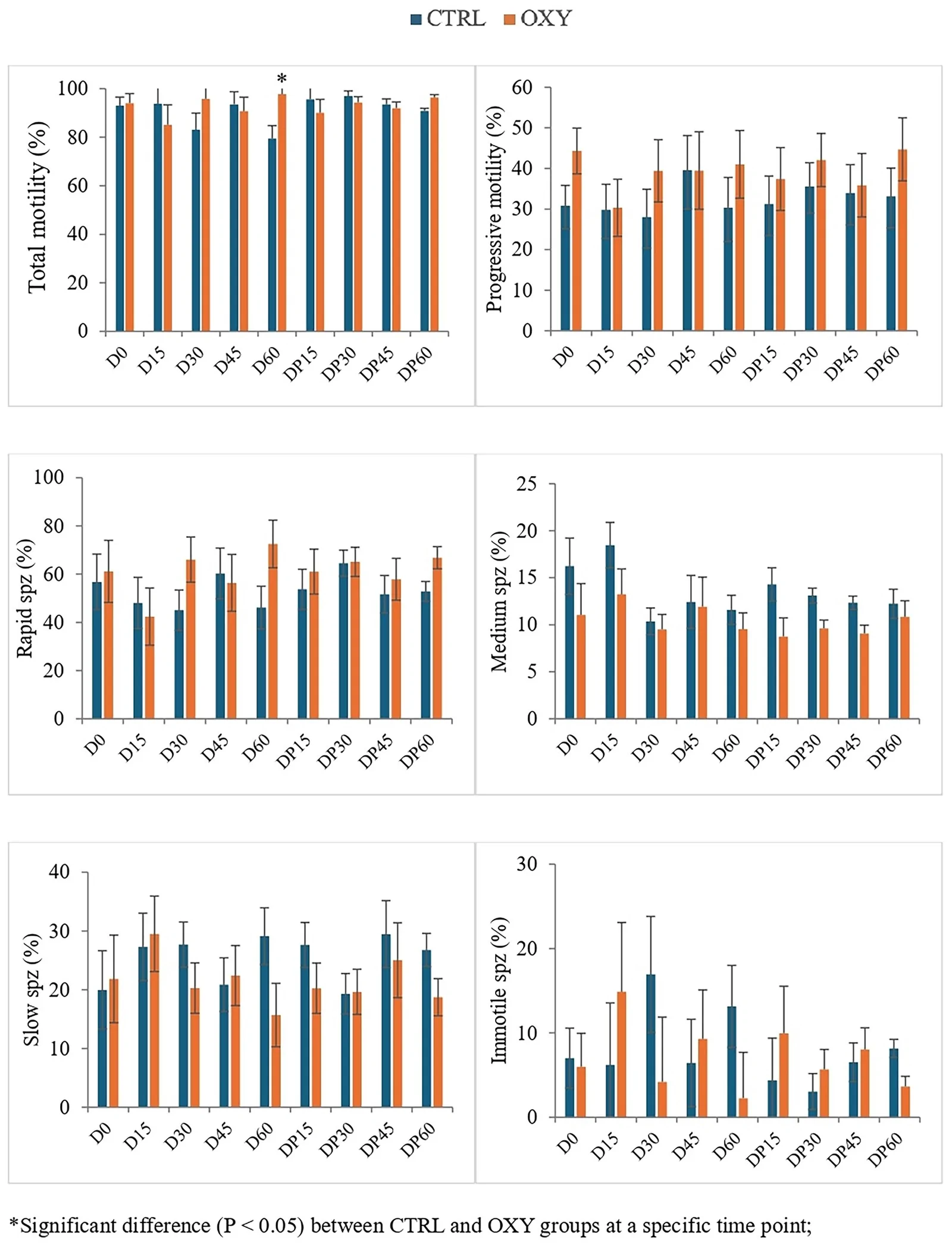
Percentage of sperm total motility, sperm progressive motility, sperm subpopulations (rapid, medium, slow, and immotile spermatozoa) in the control (CTRL) and treated (OXY) groups at different evaluation times in stallions. Data are expressed as mean ± SEM.

The results for total and progressive motility, as well as sperm subpopulations (rapid, medium, slow, and immotile), are shown in Figure 2.

Total motility differed substantially between the two treatment groups over the storage period, as demonstrated by the highly significant group × time interaction (*P* = 0.003). Specifically, post-hoc comparisons revealed that total sperm motility was significantly greater (*P* = 0.039) in the OXY group compared to the CTRL group at D60 (non-transformed mean difference: 16.19 ± 7.51%).

Furthermore, a significant group × time interaction for both the percentage of immotile spermatozoa (*P* = 0.047) was found, confirming that the temporal trajectories of this sperm subpopulation was distinctly modulated by the treatment, even though subsequent post-hoc tests did not resolve statistically significant differences at any single specific time point. No significant differences were detected in other sperm subpopulations and sperm kinetic parameters between groups (Table S3).

### Oxidative status

**Figure 3:**
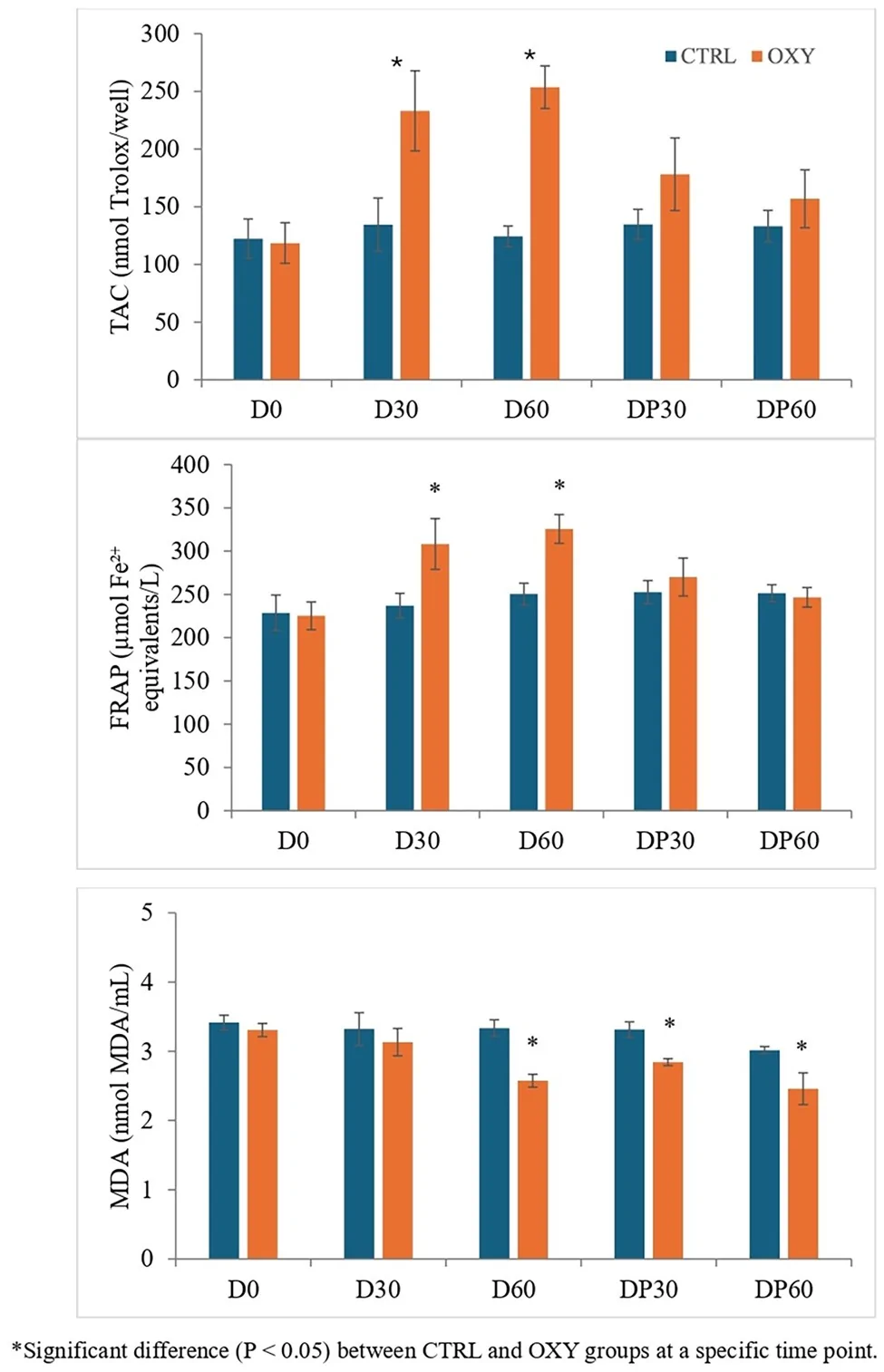
TAC, FRAP assay, and concentrations of MDA in seminal plasma of stallions at different time points in the control (CTRL) and treated (OXY) groups. Data are expressed as mean ± SEM.

TAC, FRAP values, and MDA levels in seminal plasma are shown in Figure 3. No significant main effect of group or time was observed (*P* > 0.05), whereas a significant Group × Time interaction was detected for all OS parameters (*P* = 0.031, *P* = 0.044, *P* = 0.048, respectively), indicating a treatment-dependent temporal response. Compared to the CTRL group, the OXY group exhibited greater TAC values at D30 (raw mean differences: 104.4 ± 29.79 nmol Trolox/well; *P *= 0.004) and D60 (raw mean differences: 130.34 ± 29.95 nmol Trolox/well; *P *= 0.001). Similarly, FRAP levels were greater in the OXY group at D30 (raw mean differences: 71.30 ± 24.64 µmol Fe²⁺ equivalents/L; *P* = 0.012) and D60 (raw mean differences: 75.22 ± 24.64 µmol Fe²⁺ equivalents/L; *P* = 0.009). Conversely, MDA were lower in the OXY group at D60 (raw mean differences: 0.76 ± 0.21; *P* < 0.001), DP30 (raw mean differences: 0.47 ± 0.21 nmol MDA/mL; *P* = 0.033) and DP60 (raw mean differences: 0.056 ± 0.21 nmol MDA/mL; *P* = 0.003).

## Discussion

The present study investigated the effect of a 60-d dietary supplementation with a commercial antioxidant formulation on semen quality and OS biomarkers in stallions, including an extended post-treatment follow-up. Overall, the results suggest that this specific multi-antioxidant supplementation was associated with improvements in parameters related to sperm morphology, motility and seminal plasma redox balance, with beneficial effects persisting after the end of supplementation. These findings indicate that antioxidant supplementation may exert both immediate and potentially long-lasting effects on sperm quality, likely by modulating OS during spermatogenesis and sperm maturation.

In the present study, Oxyliver supplementation did not affect semen volume and sperm concentration, in agreement with previous studies reporting that dietary antioxidants do not necessarily influence spermatogenic output or accessory gland secretions in stallions (Deichsel et al. 2008; Contri et al. 2011). These parameters are mainly regulated by endocrine and anatomical factors and appear to be less sensitive to OS than functional and structural sperm traits. Indeed, only natural antioxidants capable of modulating testosterone production have been shown to improve total sperm output (Del Prete et al. 2018). The absence of changes suggests that Oxyliver supplementation may not significantly influence the endocrine pathways involved in sperm production, which could imply limited effectiveness in cases of low sperm production or in improving libido, although further studies are needed to confirm this. However, this finding suggests that the supplementation does not markedly influence spermatogenic output or accessory gland function. In contrast, sperm functional parameters showed a greater sensitivity to antioxidant treatment. Sperm membrane functionality, assessed by the HOS test, was significantly improved with antioxidant supplementation at D60, suggesting a transient protective effect on sperm plasma membrane stability. Membrane integrity is particularly vulnerable to OS due to the high content of PUFA in stallion spermatozoa (García et al. 2011). However, this effect was not maintained at subsequent time points, indicating that antioxidant supplementation may have contributed to short-term stabilization of membrane functionality rather than a sustained effect. Similar protective effects on sperm membranes have been reported in previous studies in stallions receiving multi-antioxidant supplementation (Deichsel et al. 2008; Puerta et al. 2024).

Notably, the treated group showed a significant reduction in total sperm abnormalities and tail defects, from D60 of supplementation onward. A high proportion of morphologically normal sperm has been associated with increased fertility in stallions, while sperm morphological abnormalities are linked to reduced pregnancy rates (Jasko et al. 1990; Love 2011). The improvement observed in this study after antioxidant supplementation, which remained consistent up to 60 d post-supplementation, represents one of the most relevant findings in our study. Sperm morphological abnormalities such as tail defects and retained cytoplasmic droplets are widely recognized as indicators of impaired spermiogenesis and incomplete epididymal maturation (Amann 2010). These defects are often closely associated with OS, which may disrupt the tightly regulated processes of chromatin condensation, flagellar assembly, and cytoplasmic extrusion during spermatid differentiation (Aitken and Baker 2006). Excessive ROS can potentially damage membrane lipids, proteins, and DNA, leading to structural anomalies that compromise sperm function (Agarwal et al. 2014). Moreover, incomplete epididymal maturation may prevent the normal remodeling of the sperm membrane and removal of residual cytoplasm, further contributing to morphological abnormalities (Cooper 2011). Thus, the reduction of these defects in the treated group may reflect a protective effect of antioxidant supplementation in preserving both spermatogenic integrity and post-testicular maturation processes.

The timing of the sperm morphology improvement is biologically consistent with the duration of the spermatogenic cycle in the stallion, approximately 57–58 d (Johnson et al. 1997). The multi-antioxidant supplementation may contribute to the modulation of the microenvironment of the seminiferous tubules throughout all stages of germ cell development, as well as during epididymal maturation. Comparable improvements in sperm morphology after antioxidant and nutraceutical supplementation have been previously described (Contri et al. 2011; Vélez et al. 2023), although the persistence of these effects was not evaluated. The persistence of improved sperm morphology 60 d post-supplementation represents a novel finding, suggesting a potential carry-over effect rather than demonstrating a direct causal relationship. Considering that spermatogenesis in stallions is followed by epididymal transit, which contributes to sperm maturation (Rodriguez and Bustos-Obregón 1994; Varner et al. 2015), the sperm evaluated 60 d after the end of supplementation may originate from germ cells that were at different developmental stages during the treatment period. Therefore, Oxyliver may have contributed to enhanced resistance to OS across multiple phases of sperm development, including both testicular and post-testicular maturation processes, rather than conferring protection limited to a single stage. A positive effect of Oxyliver was also recorded on sperm motility, which is considered a key indicator of sperm functional competence (Vijayaraghavan 2009), although this effect appeared to be limited and restricted to specific motility traits. Particularly, sperm treatment with Oxyliver increased total motility at the end of supplementation (D60), whereas no effect on progressive motility was observed. The lack of improvement in progressive motility, a commonly used indicator of sperm functional performance, suggests that antioxidant supplementation did not induce a generalized enhancement of sperm motility characteristics. OS has been associated with impaired sperm motility through alterations in cellular energy availability and increased structural damage (Sengupta et al. 2024). Accordingly, the shift toward a higher proportion of motile spermatozoa observed in this study may reflect a partial modulation of sperm motility characteristics after antioxidant supplementation, rather than specific modification in flagellar kinetics. Consistent with this interpretation, CASA-derived kinetic parameters were not affected, in line with previous findings in stallions showing variable responses to dietary antioxidant supplementation (Contri et al. 2011; Puerta et al. 2024). In this context, the modulation of OS biomarkers in seminal plasma provides a plausible mechanistic explanation that may be associated with the overall improvement in sperm quality after this supplementation. In this study, antioxidant supplementation significantly increased seminal antioxidant status, as assessed by TAC and FRAP assays, during the treatment period, indicating an enhancement of seminal plasma redox buffering capacity. Reduced levels of TAC have been associated with impairment of human sperm motility and morphology (Pahune et al. 2013). The observed increase in TAC, which reflects the synergistic interaction between enzymatic and non-enzymatic antioxidants in seminal plasma, at 30 and 60 d of dietary supplementation, may suggest an enhanced scavenging capacity, potentially contributing to the protection of spermatozoa from OS. The increase in FRAP values, which reflect non-enzymatic antioxidant activity, in the treated group with an increased availability of reducing molecules in seminal fluid after 30 and 60 d of supplementation. Evidence from human studies has shown an association between vitamin intake and systemic antioxidant status or antioxidant concentrations in seminal plasma (Williams et al. 2005; Mendiola et al. 2009; Lazzarino et al. 2019).

However, the transfer of antioxidants from blood to seminal plasma is selectively regulated (Lazzarino et al. 2019). The use of nutritional supplementation, especially antioxidants, is considered a valuable strategy to improve sperm quality and reduce cryodamage due to equine semen manipulation (Bazzano et al. 2021; Ullah et al. 2025). In this context, further studies are warranted to better elucidate how such supplementation influences sperm resilience during processing.

In the present study, the improved antioxidant capacity of seminal plasma may have contributed to the neutralization of ROS, potentially supporting the preservation of sperm membrane integrity from lipid peroxidation (Barati et al. 2020). This was supported by a marked and progressive reduction in MDA concentrations, reflecting decreased lipid peroxidation. Although TAC and FRAP values showed a gradual decline over time, they remained consistent with an overall enhanced antioxidant status, suggesting a protective effect. This apparent dissociation may suggest that antioxidant supplementation not only increased the immediate antioxidant defenses of seminal plasma but also may have influenced the susceptibility of newly produced spermatozoa to oxidative damage. The sustained reduction in lipid peroxidation is particularly relevant, as oxidative damage to membrane lipids is a major determinant of sperm dysfunction in stallions (García et al. 2011; Peña et al. 2019). The persistence of lower MDA levels after supplementation withdrawal may support the hypothesis that antioxidant intake exerts its primary effects during spermatogenesis, although a causal relationship cannot be established within the present study design. Similar reductions in lipid peroxidation after antioxidant supplementation have been reported in stallions (Contri et al. 2011; Puerta et al. 2024), but the long-term persistence of this effect has not been previously demonstrated.

Furthermore, the TBARS assay’s analytical limitations should be considered when interpreting these results. Although extensively employed as an indirect indicator of lipid peroxidation, the reaction is not chemically specific to MDA because TBA can react with other aldehydes and carbonyl-containing substances. Nonetheless, because all samples were collected, maintained, and analyzed in standardized settings, the test remains useful for comparing experimental timepoints, especially when combined with TAC and FRAP. More selective techniques, such as HPLC- or LC–MS-based MDA quantification, are warranted to confirm these observations in future studies.

The observed effects may be related to the combined composition of Oxyliver, although the contribution of individual components or potential synergistic interactions cannot be determined from the present study design. Antioxidant biomarker data suggest that antioxidant compounds present in Oxyliver may be absorbed and subsequently distributed to the reproductive tract. The combination of enzymatic antioxidants, such as SOD derived from melon extract, and non-enzymatic antioxidants, including vitamins C and E, may allow complementary scavenging of ROS at different stages of oxidative pathways (Bouhadana et al. 2025). In addition, it should be noted that silybin has been reported to contribute to antioxidant defense through multiple mechanisms, including direct free radical scavenging, inhibition of their formation, and the regulation of cellular redox balance through the activation of endogenous antioxidant systems (Surai 2015; Cai et al. 2024). The upregulation of the antioxidant systems, particularly glutathione peroxidase and SOD activities, via Nrf2 signaling pathways, has been described in other models but was not evaluated in the present study. In this context, such mechanisms have been suggested to contribute to a cellular resilience to OS that may persist beyond the active administration period (Surai 2015; Cai et al. 2024). Furthermore, curcumin supplementation, which is also present in this supplement, has been shown in rat models to reduce testicular OS and minimize sperm morphological abnormalities (Takhtfooladi et al. 2015; Lonare et al. 2016). This multifaceted antioxidant approach is hypothesized to support a potential synergistic effect, which might contribute to the durability of the observed effects. However, since our study design did not include a head-to-head comparison with a single-antioxidant supplementation, this synergy hypothesis remains untested and requires further comparative investigation. Similarly, the proposed “carry-over” effect may reflect a sustained modulation of the redox balance of the reproductive tract or stabilization of the blood-testis barrier (Margret and Jain 2024), thereby providing prolonged protection against OS. These findings may have practical implications for stud farm management, although further studies are needed to confirm their applicability under field conditions. A single 60-d treatment cycle may provide antioxidant support extending beyond the supplementation period, which could be of practical interest for breeding management. However, its effectiveness across the entire breeding season was not assessed and requires further investigation.

The limitations of this study are that the experimental population was relatively small and included different breeds with a wide age range, which may limit the direct generalizability of our findings to the broader stallion population. However, the selection of this cohort was strictly dictated by the requirement of absolute environmental and management uniformity; indeed, all stallions were housed in the same breeding center under identical dietary, housing, and handling conditions. This setup aimed to reduce confounding variables, such as differences in nutrition or housing, that can affect semen quality and OS biomarkers, thereby strengthening the reliability and consistency of data. Further multi-center studies with larger cohorts will be valuable to confirm these findings on a wider scale.

In conclusion, this study demonstrated that a 60-d dietary supplementation with this specific combined antioxidant formulation improved seminal plasma redox balance, reduced lipid peroxidation, and positively modulated selected sperm quality parameters in stallions, with several benefits persisting beyond the supplementation period. These findings suggest that the tested combined antioxidant products may represent a nutritional strategy to mitigate OS and improve semen quality in breeding stallions, although their effects on reproductive performance remain to be established. However, given the limited sample size and the absence of direct fertility outcomes, these results should be interpreted with caution. Future studies involving larger cohorts, different management conditions, and fertility assessments are needed to confirm these findings and to compare multi-ingredient formulations directly with single-compound supplementations to clarify their relative efficacy and mechanisms of action.

## Supplementary Material

skag265_Supplementary_Data

## Supplementary data

*Conflicts of interest statement*. The authors declare that they have no known competing financial interests or personal relationships that could have appeared to influence the work reported in this paper. The commercial supplement used in this study is a commercially available product provided by Candioli Pharma (Beinasco, Torino, Italy), who also provided the technical information regarding its composition. Candioli Pharma had no role in the study design, data collection, statistical analysis, interpretation of data, writing of the manuscript, or the decision to submit the paper for publication.

## Abbreviations:

ADF: acid detergent fiber
ADL: acid detergent lignin
ALH: amplitude of lateral head displacement
ANOVA: analysis of variance
BCF: beat cross frequency
CP: crude protein
CTRL: control group
DM: dry matter
EE: ether extract
FRAP: ferric reducing/antioxidant power
HOS: hypoosmotic swelling test
IU: international units
LIN: linearity
MDA: malondialdehyde
NDF: neutral detergent fiber
OS: oxidative stress
OXY: treated group
PUFA: polyunsaturated fatty acids
ROS: reactive oxygen species
SCA: sperm class analyzer
SE: standard error
SOD: superoxide dismutase
SPSS: Statistical Package for the Social Sciences
STR: straightness
TAC: total antioxidant capacity
VAP: average path velocity
VCL: curvilinear velocity
VSL: straight-line velocity
