# Supplementary material for: Sixty-day oral antioxidant supplementation enhances sperm morphology and seminal plasma antioxidant capacity in stallions
Source: J Anim Sci. 2026 Aug 25;104:skag265. doi: 10.1093/jas/skag265 (PMC13570791; doi:10.1093/jas/skag265)
Supplement: Supplementary file 1 [file skag265_supplementary_data.docx]

**Supplementary Table 1.** Volume and concentration of stallion semen in control (CTRL) and treated (OXY) groups at different evaluation times. Data are expressed as mean ± SEM.

|  | Volume (mL) | |  | Concentration (x 10^6^ spz/mL) | |
| --- | --- | --- | --- | --- | --- |
|  | **CTRL** | **OXY** |  | **CTRL** | **OXY** |
| D0 | 72.5 ± 19.6 | 119.6 ± 19.6 |  | 169.9 ± 33.9 | 140.4 ± 33.9 |
| D15 | 88.5 ± 19.6 | 100.8 ± 20.8 |  | 244.6 ± 33.9 | 213.0 ± 37.8 |
| D30 | 58.0 ± 19.6 | 76.4 ± 19.6 |  | 208.2 ± 33.9 | 159.0 ± 33.9 |
| D45 | 57.9 ± 19.6 | 72.8 ± 19.6 |  | 222.2 ± 33.9 | 236.6 ± 33.9 |
| D60 | 52.8 ± 19.6 | 65.0 ± 19.6 |  | 213.8 ± 33.9 | 205.6 ± 33.9 |
| DP15 | 57.7 ± 19.6 | 54.5 ± 19.6 |  | 220.0 ± 33.9 | 163.6 ± 33.9 |
| DP30 | 57.9 ± 19.6 | 49.5 ± 19.6 |  | 232.8 ± 33.9 | 237.8 ± 33.9 |
| DP45 | 48.9 ± 19.6 | 38.0 ± 19.6 |  | 247.8 ± 33.9 | 178.2 ± 33.9 |
| DP60 | 62.9 ± 19.6 | 61.5 ± 19.6 |  | 256.6 ± 33.9 | 234.0 ± 33.9 |

**Supplementary Table 2.** Morphological abnormalities of stallion semen in control (CTRL) and treated (OXY) groups at different evaluation times. Data are expressed as mean ± SEM. P-values are reported when statistical differences are present between groups at each time point.

|  | Total abnormalities (%) | | significant P-value | Tail defects (%) | | significant P-value |
| --- | --- | --- | --- | --- | --- | --- |
|  | **CTRL** | **OXY** |  | **CTRL** | **OXY** |  |
| D0 | 35.4 ± 5.4 | 29.3 ± 6.0 |  | 31.2 ± 5.1 | 25.5 ± 5.7 |  |
| D15 | 39.4 ± 5.1 | 37.5 ± 5.7 |  | 36.6 ± 5.3 | 32.0 ± 5.9 |  |
| D30 | 31.6 ± 3.6 | 23.5 ± 4.1 |  | 26.4 ± 4.1 | 19.5 ± 4.5 |  |
| D45 | 31.0 ± 3.8 | 18.0 ± 4.3 |  | 24.0 ± 3.0 | 15.8 ± 3.3 |  |
| D60 | 32.4 ± 3.5 | 13.2 ± 3.9 | P=0.003 | 27.2 ± 3.3 | 12.2 ± 3.6 | P = 0.008 |
| DP15 | 31.0 ± 3.3 | 15.0 ± 3.7 | P=0.009 | 26.2 ± 3.0 | 12.5 ± 3.3 | P = 0.014 |
| DP30 | 28.2 ± 2.3 | 16.3 ± 2.5 | P=0.018 | 24.6 ± 2.2 | 13.3 ± 2.4 | P = 0.020 |
| DP45 | 27.2 ± 2.5 | 16.0 ± 2.0 |  | 25.3 ± 2.1 | 15.0 ± 2.3 |  |
| DP60 | 24.0 ± 1.6 | 14.8 ± 1.8 |  | 25.8 ± 1.6 | 14.5 ± 1.6 |  |
|  | **Midpiece abnormalities** (%) | |  | **Cytoplasmic droplets** (%) | |  |
|  | **CTRL** | **OXY** |  | **CTRL** | **OXY** |  |
| D0 | 2.2 ± 0.8 | 2.5 ± 1.3 |  | 2.0 ± 0.9 | 1.3 ± 1.0 |  |
| D15 | 2.4 ± 1.9 | 3.0 ± 1.3 |  | 0.4 ± 0.5 | 1.5 ± 0.5 |  |
| D30 | 2.4 ± 0.8 | 2.0 ± 0.9 |  | 2.8 ± 0.6 | 2.0 ± 0.7 |  |
| D45 | 2.6 ± 0.9 | 1.5 ± 1.0 |  | 2.4 ± 0.7 | 0.8 ± 0.8 |  |
| D60 | 3.8 ± 1.2 | 0.5 ± 1.4 |  | 1.4 ± 0.8 | 1.0 ± 0.9 |  |
| DP15 | 2.6 ± 1.0 | 1 ± 1.1 |  | 2.2 ± 0.9 | 1.5 ± 1.0 |  |
| DP30 | 1.8 ± 1.1 | 1.8 ± 1.2 |  | 1.8 ± 0.8 | 1.2 ± 0.8 |  |
| DP45 | 2.2 ± 0.6 | 0.5 ± 0.7 |  | 1.8 ± 0.3 | 0.5 ± 0.4 |  |
| DP60 | 1.8 ± 0.2 | 1.3 ± 0.2 |  | 2.2 ± 0.8 | 0.5 ± 1.0 |  |

**Supplementary Table 3.** Kinetic parameters of stallion semen in control (CTRL) and treated (OXY) groups at different evaluation times. Data are expressed as mean ± SEM.

|  | VCL (μm/s) | |  | VSL (μm/s) | |
| --- | --- | --- | --- | --- | --- |
|  | **CTRL** | **OXY** |  | **CTRL** | **OXY** |
| D0 | 123.3 ± 23.7 | 135.6 ± 26.6 |  | 46.3 ± 7.4 | 56.6 ± 8.3 |
| D15 | 106.0 ± 15.7 | 89.7 ± 17.6 |  | 42.7 ± 8.0 | 36.8 ± 8.9 |
| D30 | 107.0 ± 17.9 | 143.5 ± 20.0 |  | 41.2 ± 6.5 | 60.1 ± 7.3 |
| D45 | 122.0 ± 14.3 | 121.1 ± 16.0 |  | 55.7 ± 9.7 | 52.8 ±10.9 |
| D60 | 107.6 ± 14.9 | 150.4 ± 16.6 |  | 50.5 ± 9.9 | 60.8 ± 11.0 |
| DP15 | 114.0 ± 15.1 | 132.4 ± 17.0 |  | 45.9 ± 7.0 | 58.4 ± 7.8 |
| DP30 | 132.9 ± 8.7 | 130.2 ± 9.7 |  | 51.6 ± 5.4 | 56.4 ± 6.1 |
| DP45 | 109.6 ± 12.2 | 121.8 ± 13.6 |  | 45.0 ± 5.9 | 54.1 ± 6.7 |
| DP60 | 116.3 ± 7.8 | 128.3 ± 8.8 |  | 46.4 ± 7.4 | 56.4 ± 8.3 |
|  | **VAP** (μm/s) | |  | **LIN** (%) | |
|  | **CTRL** | **OXY** |  | **CTRL** | **OXY** |
| D0 | 70.3 ± 12.1 | 74.7 ± 13.5 |  | 35.3 ± 3.3 | 39.6 ± 3.7 |
| D15 | 60.1 ± 10.6 | 51.2 ± 11.9 |  | 34.1 ± 3.4 | 33.7 ± 3.8 |
| D30 | 59.0 ± 9.0 | 82.7 ± 10.1 |  | 35.1 ± 4.3 | 40.7 ± 4.8 |
| D45 | 76.8 ± 10.1 | 70.3 ± 11.3 |  | 43.7 ± 4.7 | 40.0 ± 5.3 |
| D60 | 57.1 ± 7.2 | 85.6 ± 8.0 |  | 39.3 ± 3.8 | 38.2 ± 4.2 |
| DP15 | 67.9 ± 8.0 | 79.5 ± 8.9 |  | 40.6 ± 5.0 | 42.1 ± 5.6 |
| DP30 | 77.1 ± 4.3 | 75.2 ± 4.8 |  | 37.5 ± 4.4 | 40.0 ± 5.0 |
| DP45 | 63.5 ± 6.3 | 73.8 ± 7.0 |  | 39.9 ± 5.1 | 41.6 ± 5.7 |
| DP60 | 67.6 ± 6.4 | 74.7 ± 7.1 |  | 37.3 ± 4.9 | 40.3 ± 5.5 |
|  | **STR** (%) | |  | **ALH** (μm) | |
|  | **CTRL** | **OXY** |  | **CTRL** | **OXY** |
| D0 | 60.6 ± 2.6 | 67.6 ± 2.9 |  | 2.6 ± 0.5 | 2.8 ± 0.5 |
| D15 | 60.2 ± 2.2 | 60.7 ± 2.5 |  | 2.4 ± 0.3 | 2.1 ± 0.3 |
| D30 | 62.7 ± 3.4 | 66.4 ± 3.8 |  | 2.3 ± 0.4 | 2.9 ± 0.4 |
| D45 | 66.8 ± 3.3 | 67.3 ± 3.7 |  | 2.4 ± 0.3 | 2.5 ± 0.3 |
| D60 | 64.6 ± 3.2 | 64.9 ± 3.5 |  | 2.3 ± 0.3 | 3.0 ± 0.3 |
| DP15 | 63.8 ± 3.9 | 67.1 ± 4.4 |  | 2.3 ± 0.4 | 2.6 ± 0.4 |
| DP30 | 61.1 ± 3.5 | 66.7 ± 3.9 |  | 2.7 ± 0.3 | 2.7 ± 0.3 |
| DP45 | 64.4 ± 4.4 | 65.7 ± 4.9 |  | 2.3 ± 0.3 | 2.4 ± 0.3 |
| DP60 | 61.1 ± 4.1 | 67.2 ± 4.6 |  | 2.4 ± 0.2 | 2.6 ± 0.2 |
|  | **BCF** (beats/s) | |  | **Hyperactive** (%) | |
|  | **CTRL** | **OXY** |  | **CTRL** | **OXY** |
| D0 | 15.1±1.4 | 17.6±1.6 |  | 22.8±9.3 | 24.6±10.4 |
| D15 | 11.4±1.9 | 10.9±2.1 |  | 16.7±3.6 | 11.8±4.0 |
| D30 | 14.8±2.0 | 17.9±2.3 |  | 17.6±7.6 | 24.0±8.5 |
| D45 | 16.5±1.9 | 17.4±2.1 |  | 14.4±7.1 | 18.7±8.0 |
| D60 | 14.4 ± 1.9 | 20.5 ± 2.1 |  | 15.4 ± 6.5 | 27.5 ± 7.3 |
| DP15 | 15.6 ± 1.5 | 18.4 ± 1.7 |  | 16.4 ± 7.0 | 18.8 ± 7.9 |
| DP30 | 17.6 ± 1.1 | 17.9 ± 1.2 |  | 22.2 ± 6.9 | 21.7 ± 7.7 |
| DP45 | 14.9 ± 1.6 | 16.8 ± 1.7 |  | 17.0 ± 5.6 | 16.0 ± 6.2 |
| DP60 | 15.4 ± 1.2 | 18.7 ± 1.3 |  | 16.8 ± 5.1 | 19.2 ± 5.7 |

VCL = curvilinear velocity; VSL = straight line velocity; VAP = average path velocity; LIN = linearity; STR = straightness coefficient; ALH = amplitude of lateral head displacement; BCF = beat/cross frequency.
